# The effect of a new perioperative practice model on length of hospital stay and on the surgical care process in patients undergoing hip and knee arthroplasty under spinal anesthesia: a randomized clinical trial

**DOI:** 10.1186/s12912-020-00465-3

**Published:** 2020-07-31

**Authors:** Maria Pulkkinen, Irma Jousela, Janne Engblom, Sanna Salanterä, Kristiina Junttila

**Affiliations:** 1 University of Helsinki, Helsinki University Hospital, Perioperative, Intensive Care and Pain Medicine, PO. Box. 900, Vantaa, FI00029 Helsinki, HUS Finland; 2 Department of Nursing Science, University of Turku, Joukahaisenkatu 3-5, 20520 Turku, Finland; 3 University of Eastern Finland, Kuopio, Finland; 4 Department of Mathematics and Statistics, University of Turku, Turku, Finland; 5 School of Economics, University of Turku, Rehtorinpellonkatu 3, 20500 Turku, Finland; 6 Turku University Hospital, Kiinanmyllynkatu 4-8, 20500 Turku, Finland; 7 University of Helsinki and Helsinki University Hospital, Nursing Research Center, Tukholmankatu 8F, PO.Box. 442, FI00029 Helsinki, HUS Finland

**Keywords:** Arthroplasty, LOS, New perioperative practice model, Surgical care process

## Abstract

**Background:**

The shortened length of hospital stays (LOS) requires efficient and patient-participatory perioperative nursing approaches to enable early and safe discharge from hospitals for patients undergoing total hip arthroplasty (THA) and total knee arthroplasty (TKA). The primary aim of this study was to explore the effect comparative to standard perioperative care of a new perioperative practice model (NPPM) on the LOS and the time points of the surgical care process in patients undergoing THA and TKA under spinal anesthesia. The secondary aim was to find out if any subgroups with different response could be found.

**Methods:**

Patients scheduled for elective, primary THA and TKA were assessed for eligibility. A two-group parallel randomized clinical trial was conducted with an intervention group (*n* = 230) and control group (*n* = 220), totaling 450 patients. The patients in the intervention group were each designated with one named anesthesia nurse, who took care of the patient during the entire perioperative process and visited the patient postoperatively. The patients in the control group received standard perioperative care from different nurses during their perioperative processes and without postoperative visits. The surgical care process time points for each study patient were gathered from the operating room management software and hospital information system until hospital discharge.

**Results:**

We did not find any statistically significant differences between the intervention and control groups regarding to LOS. Only slight differences in the time points of the surgical care process could be detected. The subgroup examination revealed that higher age, type of arthroplasty and ASA score 3–4 all separately caused prolonged LOS.

**Conclusion:**

We did not find the new perioperative practice model to shorten either length of hospital stays or the surgical care process in patients undergoing THA and TKA. Further studies at the subgroup level (gender, old age, and ASA score 3 and 4) are needed to recognize the patients who might benefit most from the NPPM.

**Trial registration:**

This study was registered in NIH Clinical.Trials.gov under registration number NCT02906033, retrospectively registered September 19, 2016.

## Background

Primary total hip arthroplasty (THA) and total knee arthroplasty (TKA) are among the most common surgical procedures performed nowadays. Both procedures usually result in improvements to mobilization and overall quality of life for the patients [1, 2]. Fast-track protocols and different kinds of early recovery programs have been implemented that have shortened LOS following THA and TKA procedures [3–6]. In some institutions, THA and TKA are already being performed as day surgery procedures in select cases with promising results. According to previous studies, shortened LOS requires effective, patient-centered, patient-activated interventions to motivate patients to be active participants in their own care. These strategies can facilitate early discharge for arthroplasty patients [7–10].

Most patients recover very fast which allows discharge from the hospital the second day after THA and the third day after TKA [11]. But some patients still seem to recover much more slowly than others, barely managing to leave the hospital one week following arthroplasty [12] and requiring more support, individual education and guidance in pain management pertaining to self-care. These patients could benefit from even more participatory and individual care activities provided by perioperative nurses. There is a need to implement innovative perioperative nurse interventions for decreased LOS.

The current demands for efficiency in the operating room (OR) require multidisciplinary cooperation throughout the perioperative process to ensure patients undergoing THA and TKA are feeling confident and safe at their early discharge from the hospital. Perioperative nurses have a key-role in the multidisciplinary team, coordinating the surgical patient care in right direction [5, 11]. In Finland, the term ‘perioperative nursing’ is used despite the nurses’ job description in the operating department. Therefore, an anesthesia nurse (AN) is titled perioperative nurse.

There are differences between countries in the scope of practice of an anesthesia nurse (AN). Most ANs have specialist training in anesthesia nursing after their registered nurse education for about two and a half year. The title AN is not the same as nurse anesthetist. While the nurse anesthetist is permitted to administer anesthesia, the AN is not [13, 14]. In Finland, an AN works together with the anesthetist. The anesthetist intubates and extubates the patient and applies spinal and epidural anesthesia, which are not included in the practice scope of an AN [15]. The anesthetist is present only in these fore mentioned situations, and in case there is something emerging occurring. The AN maintains anesthesia according to the anesthetist’s prescriptions and takes care of the patient by supporting the patient both psychologically and physiologically during the operation. Patient information and education about pain management, exercise, and mobilization are provided by the AN already in the OR. Patient education is a professional duty of healthcare professionals in the Nordic countries and stated in the rights of the patient [16].

The impact of preoperative education has been reported to be of great importance for patients undergoing THA or TKA [17]. It has been reported that these patients do not receive as much knowledge as they wish to receive, and this might influence patient satisfaction [18]. The preoperative education and information have been reported to engage and empower patients to selfcare and thereby enhance their recovery, this could be essential in the light of ever shortening LOS. The preoperative patient education and information prepares the patients for surgery and further for the recovery [19]. All patients suffer from anxiety to some degree prior to surgery and patients at poor emotional state preoperatively have been reported to have poorer outcomes resulting in prolonged LOS. Anxiety has been reported to diminish while the patient receives adequate education and information [20].

The authors tested a new perioperative practice model (NPPM) in a pilot study with a qualitative approach involving 20 THA and TKA patients. The purpose of the pilot study was to describe how patients undergoing THA or TKA experienced to be cared for by the one and same AN during the entire perioperative process. The patients included in the pilot study found this intervention greatly beneficial. The findings showed that patients experienced they were met with respect and the nurses took their concerns seriously. They felt involved and safe in their own care, and they experienced the continuity created by the same nurse as crucial while they did not have to tell their story repeatedly to several nurses. The emotional support, trust and encouragement provided by NPPM was experienced as vital by the participants [21]. The promising findings of the pilot study needed to be carefully studied in this randomized clinical trial. Continuity of care delivered by the same AN may help reduce anxiety and improve patient experience.

In the NPPM the patient-nurse relationship is a continuous relationship, where the patient had his/her own designated AN throughout the perioperative process. This was expected to create trust and comfort to the patient and promote self-care. Factors influencing interruptions in the pain management and coordination of care were avoided because there were no handovers between different nurses at transition from the OR to the post-anesthesia care unit (PACU).

In this study we hypothesized that the intervention group would have statistically significantly shorter mean LOS than the control group. To our knowledge there is limited research that examines how perioperative nurse delivered interventions influence the LOS in patients undergoing THA and TKA.

## Methods

### Aim

The primary aim of this study was to explore the effect of a new perioperative practice model (NPPM) on the LOS and the time points of the surgical care process in patients undergoing THA and TKA under spinal anesthesia, compared to those of patients in standard perioperative care. The secondary aim of this study was to find out if any subgroups with different response could be found.

### Study design

The study was a two-group parallel single-blind randomized clinical trial.

### Sample and setting

The study was conducted at Peijas Hospital, which is a high-volume center for total joint arthroplasties and revision arthroplasties at Helsinki University Hospital in Finland. The unit has a fast-track program in use consisting of early mobilization on the day of surgery, aggressive physiotherapy, opioid-sparing analgesia and patient education provided to all THA and TKA patients preoperatively. The hospital maintains established discharge criteria for THA and TKA patients.

**Fig. 1:**
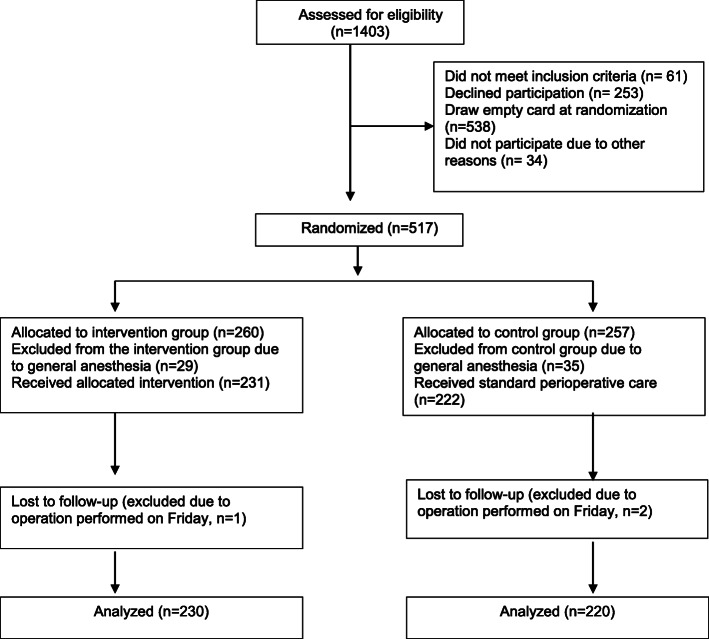
Flow chart of the study participants and reasons for exclusion and dropouts

The study sample consisted of adult female and male patients scheduled for primary THA and primary TKA. The participants were recruited at their preoperative visits to the outpatient clinic 2–3 weeks prior to their scheduled operations. The inclusion criteria were that the participants were 18 years of age or older, that the operations were planned to be performed under spinal anesthesia and scheduled between Monday and Thursday, and that the participants comprehended the research information. Reasons for exclusion and dropout of participants are presented in the flow chart of the study (Fig. 1).

### Randomization

Randomization was performed by an independent third party (a nurse at the outpatient clinic during the preoperative visit 2–3 weeks prior to scheduled operation). Eligible patients drew one of two cards; one indicated invitation to participate the study and the other was blank indicating not participating in the study. Patients for the control group and the intervention group were by the group both recruited and scheduled for operation every other week. This stratification aimed to ensure that the patients in the two groups did not exchange information in the postoperative ward. Furthermore, the patients receiving intervention had their own AN visiting them postoperatively and the control group patients did not. This could have been experienced as unfair or even un-ethical by the patients in the control group. The participants randomized for the study were masked. The designated anesthesia nurses were not masked due to the nature of the interventions. The recruitment of patients started in September 2016 and was completed in December 2017.

### Intervention group (NPPM care)

All patients in the intervention group had a designated AN during their entire perioperative processes (care preoperative, in the OR and in the PACU). The same designated AN visited his or her patient the next day after surgery at the ward. Actual nursing care in the OR and in the PACU remained the same as in standard perioperative care. The focus of the NPPM was on the patients’ personal and individual care needs, and it enabled emotional support, motivation to participate in self-care and continuity of care, as the ANs remained the same during the entire perioperative processes. General information about the study was offered to the entire nursing personnel of the operating department. There was no need for extra training of the ANs since they educate and inform all the patients as a standard praxis. The only thing that differed from ordinary care was the continuity of the care and a postoperative visit to the ward next day after surgery.

### Control group (standard perioperative care)

The patients in the control group received standard perioperative care. Under the standard, the patients were cared for by different nurses preoperatively, in both the OR and the PACU. Postoperative visits are not currently performed in the department where this study was conducted.

### Data collection

Data were collected prospectively from participants’ hospital electronic record. The surgical care process data included time points of each study patient’s care process from the operating room management software (Opera, CHCA, Quebec, Canada) and hospital information system (Uranus, CGI Finland Oy, Helsinki, Finland), tracking patient movement from admission to the hospital until discharge from it. The care process time points that were estimated were as follows: *preparation time* from the patient’s arrival to the operating department to the administration of anesthesia agents (h), *surgery time* from incision to closing of the wound (h), *operating room time* from patient entrance to the operating room to patient exit from it (h), *PACU time* (h), *recovery time* from patient readiness for discharge from PACU to patient discharge from the hospital (h) and *LOS* from hospital admission to hospital discharge (days). The demographic data collected from the participants were as follows: age, gender, procedure, American Society of Anesthesiologists (ASA) score and weekday of operation. The ASA classification consists of six scores ranging from one to six. ASA 1, a normal healthy patient, ASA 2 a patient with mild systemic disease, ASA 3 a patient with severe systemic disease, ASA 4 a patient with severe systemic disease that is a constant threat to life, ASA 5 a moribund patient who is not expected to survive without operation, ASA 6 a declared brain-dead patient whose organs are being removed for donor purpose [22].

### Data analysis

No subgroups were fixed at the time of randomization. The potential subgroups were tested in the post hoc analysis for gender (female vs. male), type of arthroplasty (THA vs. TKA), ASA score (1 and 2 vs. 3 and 4), age, weekday of operation (Monday–Tuesday vs. Wednesday–Thursday) and LOS ≤ 3 days vs. LOS >  3 days. We used the per-protocol analysis in our study. The patients who had a last-minute change in their anesthesia type (general anesthesia) were excluded in both groups because this was designed to be a major deviation of the protocol.

Descriptive statistics were used to present characteristics of the study participants. Multi-factor ANOVA was used to compare means of the groups. Interaction terms were used to compare means of the groups defined by combination of categorical independents. Statistical analysis was performed using SAS® version 9.4.

### Sample size and statistical methods

The sample size requirement for comparing two LOS means was checked with power analysis (2-sided test) with α = 0.05, β = 0.9, standard deviation = 1.6 and differences of means = 0.5 days. Sufficient sample size was determined to be *n* = 217 patients per group. For *PACU time*, it was determined as *n* = 76 patients per group (standard deviation = 0.94, differences of means = 0.5 h).

## Results

The final sample size was 220 participants in the control group and 230 in the intervention group, in total 450 patients. Out of the 450 participants 63% (*n* = 282) were females. The age of the participants ranged from 29 to 92 years (mean 67 years SD 10.44). The sample characteristics are presented in Table 1. There were no significant differences between the intervention and control groups at baseline.

**Table 1:** Characteristics of the study participants (*n* = 450)

	Intervention group	Control group	Total
**Gender, n (%)**			
Male	85 (37.0)	83 (37.7)	168
Female	145 (63.0)	137 (62.3)	282
**Age (mean, SD)**	67 (10.41)	68 (10.48)	67 (10.44)
**Type of operation, n (%)**			
THA	143 (62.2)	137 (62.3)	280
TKA	87 (37.8)	83 (37.7)	170
**ASA score, n (%)**			
ASA 1	30 (13.0)	32 (14.6)	62
ASA2	102 (44.4)	107 (48.6)	209
ASA 3–4	98 (42.6)	81 (36.8)	179

The mean *LOS* (days) was 3.08 in the intervention group and 3.18 in the control group (difference of means = − 0.10, 95% CI [− 040, 0.19] *p* = 0.49). Converted to hours, the mean LOS was 2.40 h shorter in the intervention group. There was a statistically significant difference between the control group and the intervention group in the *surgery time* (difference of means = 0.09, 95% CI [0.01, 0.17] *p* = 0.02). When converted to minutes, the mean surgery time was 5 min shorter in the control group. A statistically significant difference was also found in the mean *operating room time* between the intervention group (2.65 h) and the control group (2.52 h) (difference of means = 0.12, 95% CI [0.02, 0.22] *p* = 0.01). Converted to minutes, the mean operating room time was 7 min shorter in the control group. The results are presented in Table 2.

**Table 2:** Differences of means, 95% CI and *p*-values in the LOS, PACU time and the length of total surgical process between the intervention group and the control group, gender within the groups and the surgical procedures total hip arthroplasty (THA) and total knee arthroplasty (TKA). Total amount of patients n = 450

***Preparation time*** from patient’s arrival to the operating department to administration of anesthesia agents						
	Hours		Hours	Difference of means	95% CI	***p***-value
**Intervention group all**	**0.35**	**Control group all**	**0.36**	**−0.01**	**[−0.03, 0.01]**	**0.52**
Female THA	0.36	Female THA	0.37	−0.00	[−0.07, 0.05]	0.99
Male THA	0.35	Male THA	0.35	0.00	[−0.07, 0.07]	1.00
Female TKA	0.37	Female TKA	0.38	−0.00	[− 0.08, 0.06]	0.99
Male TKA	0.32	Male TKA	0.33	−0.00	[−0.11, 0.09]	1.00
***Surgery time*from incision to closing of the wound**						
**Intervention group all**	**1.53**	**Control group all**	**1.43**	**0.09**	**[0.01, 0.17]**	**0.02**
Female THA	1.47	Female THA	1.41	0.06	[−0.13, 0.26]	0.98
Male THA	1.43	Male THA	1.41	0.02	[−0.22, 0.26]	1.00
Female TKA	1.47	Female TKA	1.44	0.02	[−0.21, 0.27]	1.00
Male TKA	1.84	Male TKA	1.38	0.45	[0.09, 0.81]	0.00
***Operating room time*from patient entrance to operating room to patient exit from it**						
**Intervention group all**	**2.65**	**Control group all**	**2.52**	**0.12**	**[0.02, 0.22]**	**0.01**
Female THA	2.62	Female THA	2.53	0.05	[−0.16, 0.34]	0.96
Male THA	2.58	Male THA	2.49	0.08	[−0.22, 0.39]	0.99
Female TKA	2.57	Female TKA	2.59	−0.01	[− 0.32, 0.29]	1.00
Male TKA	3.01	Male TKA	2.42	0.59	[0.13, 1.05]	0.00
***PACU time***						
	**Hours**		**Hours**	**Difference of means**	**95%CI**	***p*-value**
**Intervention group all**	**1.95**	**Control group all**	**2.07**	**−0.12**	**[−0.29, 0.05]**	**0.18**
Female THA	1.97	Female THA	1.99	−0.01	[− 0.45, 0.42]	1.00
Male THA	1.78	Male THA	2.21	−0.42	[−0.95, 0.10]	0.22
Female TKA	2.25	Female TKA	2.09	0.16	[−0.36, 0.68]	0.98
Male TKA	1.62	Male TKA	1.94	−0.31	[−1.09, 0.45]	0.91
***Recovery time*from patient readiness for discharge from PACU to patient discharged from the hospital**						
**Intervention group all**	**66.30**	**Control group all**	**68.88**	**−2.58**	**[−9.77, 4.61]**	**0.48**
Female THA	66.58	Female THA	66.31	0.26	[−18.02, 18.56]	1.00
Male THA	57.45	Male THA	71.89	−14.44	[−36.70, 7.81]	0.49
Female TKA	73.19	Female TKA	69.02	4.17	[−17.88, 26.22]	0.99
Male TKA	68.62	Male TKA	69.73	−1.11	[−33.63, 31.40]	1.00
***LOS*from hospital admission to hospital discharge (days)**						
**Intervention group all**	**3.08**	**Control group all**	**3.18**	**−0.10**	**[−0.40, 0.19]**	**0.49**
Female THA	3.07	Female THA	3.06	0.00	[−1.51, 0.35]	0.55
Male THA	2.71	Male THA	3.29	−0.58	[−0.76, 0.77]	1.00
Female TKA	3.41	Female TKA	3.22	0.19	[−0.73, 1.12]	0.99
Male TKA	3.16	Male TKA	3.23	−0.07	[−1.43, 1.29]	1.00

The statistically significant differences for variables associated with longer LOS were high age (mean age for LOS > 3 days 71 years vs. 64 years for LOS ≤3 days, *p*-value <.0001), type of operation (113 patients undergoing THA out of 280 had LOS > 3 days vs. 167 patients had LOS ≤3 days, p-value <.001), and ASA scores 3–4 (out of 179 patients classified as ASA 3–4, 114 patients had LOS > 3 days vs. 64 patients LOS ≤3 days, p-value <.0001). Descriptive statistics for patients with LOS ≤ 3 days vs. LOS ≥ 3 days are presented in Table 3.

**Table 3:** Descriptive statistics for patients with LOS ≤ 3 days vs. LOS ≥ 3 days

Variable	All patients	LOS ≤ 3 days	LOS > 3 days	***p***-value ≤ 3 days vs. > 3 days
Number	450	242	207	
Age, yrs., Mean (Range)	67.0 (29–92)	64.7 (36–90)	71.0 (29–92)	<.0001
Gender, n^a^ (%)				0.07
Female	282 (62.8)	143 (59.1)	139 (67.2)	
- Male	167 (37.2)	99 (40.9)	68 (32.8)	
Type of operation, n^a^ (%)				<.001
- THA	280 (62.2)	167 (69.0)	113 (54.6)	
- TKA	169 (37.6)	75 (31.0)	94 (45.4)	
ASA class, n (%)				<.0001
- ASA 1	62 (13.8)	46 (19.0)	16 (7.7)	
- ASA 2	209 (46.4)	132 (54.5)	77 (37.2)	
- ASA 3–4	179 (39.8)	64 (26.5)	114 (55.1)	
Weekday of operation, n^a^ (%)				ns.
- Monday	125 (27.8)	67 (53.6)	58 (28.0)	
Tuesday	111 (24.7)	70 (63.1)	41 (20.0)	
- Wednesday	107 (24.0)	59 (55.1)	48 (23.0)	
- Thursday	106 (23.5)	46 (43.4)	60 (29.0)	

Effective sample size = 449

^a^One frequency missing

THA Total Hip Arthroplasty

TKA Total Knee Arthroplasty

When examining the subgroups, the shortest mean *LOS* was found in male patients of the intervention group undergoing THA (mean *LOS* 2.71 days, converted to 65.04 h). In the control group the male patients undergoing THA had a mean *LOS* 3.29 days (78.96 h). The difference of means was 13 h 92 min. The shortest mean *PACU time* was in male patients in the intervention group undergoing TKA; 1.62 h (97 min). In the control group, the mean *PACU time* of the same subgroup was 1.94 h (116 min). The difference of means, 19 min, was not of statistical significance but nearly of clinical importance, which was set to be 30 min for the *PACU time*.

## Discussion

The primary aim of this study was to explore the effect of NPPM on LOS in patients undergoing THA and TKA under spinal anesthesia compared to the effect of standard perioperative nursing care. We did not find any statistically significant difference in LOS between the intervention group and control group. Although the differences between the groups were statistically significant for the *surgery time* and the *operating room time*, the differences were clinically of minor value. These differences might be explained by the fact that the LOS in our operating department was already approximately 2 days for THA patients and 3 days for TKA patients. This finding was not expected based on our pilot study and reveals the importance of having a large enough sample size in randomized controlled studies.

The secondary aim of our study was to ascertain if there were any subgroup differences that could be identified. We examined if there was a difference in length of stay following THA or TKA based on sex/gender (female/male) although stratification based on gender was not done at time of randomization.

The predictable reasons for slow recovery causing prolonged LOS has been reported as the following: older age, higher ASA score, type of arthroplasty, and gender. Older patients, especially women, have a tendency toward extended LOS [23, 24]. This might be because older patients do not have the strength and knowledge for completing standardized care programs as planned without continuous emotional support and motivation from nursing personnel; they might additionally have individual extended care needs during and after admission [25]. The findings of our study are consistent with earlier studies. In our study, 2/3 of the patients who had LOS >  3 days were female. Hustedt et al. reported the probability to stay longer than 3 days in hospital was almost 40% higher in female than in male patients [23]. Marital status was not addressed in our study, but it could be that elderly female patients might more often be widowed than male patients, due to the fact that the life expectancy of males is about six years shorter than for females [26] and thus not have spouses at home to take care of them after discharge from the hospital. LOS > 3 days was also associated with older age and higher ASA scores (3–4). In addition, patients undergoing THA had shorter LOS than patients undergoing TKA (Table 1). This finding is similar to the finding of Sutton et al. who found that patients undergoing THA were more likely to be discharged earlier than patients undergoing TKA [27].

Some studies have reported the weekday of surgery as predictive of LOS. The most critical days for surgery in THA and TKA patients have been estimated to be Thursday and Friday, which indicate prolonged LOS. This indication has been attributed to limited or lacking physiotherapist services during the weekend [28, 29]. We did not find any statistically significant differences in our study sample when it came to the day of surgery. In the sample, 53% of the patients undergoing an operation on Monday, and 63% of the patients undergoing an operation on Tuesday, had a LOS ≤ 3 days. The corresponding portions on Wednesday and Thursday were 55 and 43%, respectively. These results could be useful when planning the day of surgery for elective patients. In planning the week, it could be reasonable to schedule operations on patients suitable for day surgery on Thursday and operations on patients with high risk for prolonged LOS on either Monday, Tuesday, or Wednesday.

In reducing LOS, it might be important to identify, and target patient groups predicted to recover more slowly than others and in need of extra support, so that the right interventions can be directed preoperatively to them. According to the findings of our study, the patients who could gain from extra support and encouragement in the preoperative stage are those of older age and with ASA score 3–4 undergoing TKA. These findings are supported by a recent study [30].

To be successfully implemented, the fast-track protocol requires multidisciplinary cooperation, engagement and communication between the patient and all healthcare professionals involved in the patient’s care concerning the perioperative direction and the goals for discharge. These cooperative links can decrease LOS in patients undergoing THA and TKA [27, 31, 32]. Although patient-related factors such as medical reasons and comorbidities for prolonged LOS are known, there can still be exogenous factors based on how surgeons, anesthesiologists and perioperative nurses are practicing and communicating. There might also be the possibility that old traditions and habits among nurses are a barrier to promoting patients’ self-care [33].

The strength of our study is that we have investigated routine care in arthroplasty patients in the study setting. Since the ultimate purpose of perioperative nursing care is to promote health and well-being in surgical patients, there is a need for valid data on actual performance (patient care and outcomes) at the ordinary praxis [34]. Elective surgical care is changing rapidly, demanding new perioperative nursing interventions to fit the surgical patients’ care instead of following old traditions and habits.

One major limitation of our study might be that due to the already rather short LOS measures in our department it was almost impossible to obtain statistically significant differences of LOS between the groups. To be able to find statistically significant differences between the intervention and the control group, a larger sample size, a longer duration of the intervention and a longitudinal design could have given us different results. However, this was not possible in this study, regarding to the intervention. Another limitation in our study is that we did not recognize beforehand the subgroups on which we should have targeted the interventions and what patients really needed extra support and encouragement. The main purpose of our study was not particularly to find those who might have needed more support than others. The third limitation of our study was not addressing patient satisfaction, which could have given us valuable insight on how the patients experienced the NPPM, as patient satisfaction is a highly relevant indicator for organizational outcomes.

We did not find any statistically significant difference between the intervention and the control group. However, we found some important findings concerning male patients of the intervention group undergoing THA, their *LOS* was 13 h 92 min shorter than for male patients of the control group undergoing THA. However, this is not a statistically significant difference, but it is of clinical importance. The difference is more than the length of a working shift of a nurse (8 h) at the postoperative ward.

As a future goal of our department is to shorten the time to discharge by one day for both THA and TKA patients, the NPPM could be implemented as a part of the fast-track protocol for vulnerable patients. Responsibilities could be shared, and communication could be improved between the ward nurses, nurses of the outpatient clinic, surgeons, anesthesiologists, and perioperative nurses to enhance self-management for patients who really need it [35, 36]. Further research is required on the development of patients’ health optimization being incorporated into the fast-track pathway for THA and TKA patients. New innovative perioperative nursing interventions should be tested for this purpose, and it might be time to change old habits and traditions into new ones.

## Conclusions

This study did not find the NPPM to be superior to standard perioperative care in diminishing LOS for patients undergoing THA and TKA. Future studies are needed to examine if the NPPM model can benefit patients undergoing THA or TKA based on sex/gender, age greater than 65 years, and ASA score of 3 or higher. In clinical practice, identifying patients in need of more support and encouragement would require an instrument (e.g., a questionnaire or risk scale) to be used in advance upon a preoperative visit.

## Data Availability

The datasets generated and analyzed during the current study are not publicly available because the authors do not have the permission from the study participants to publish the collected raw data. However, after reasonable request with permission from Helsinki University Hospital authorities the data can be made available from the corresponding author.

## Abbreviations

AN: Anesthesia Nurse
ASA: American Society of Anesthesiologists Physical Status classification
LOS: Length of hospital stay
NPPM: New Perioperative Practice Model
OR: Operating Room
PACU: Post Anesthesia Care Unit
THA: Total hip arthroplasty
TKA: Total knee arthroplasty
